# Population immunity, post-vaccine and post-infection antibody responses to influenza A (H3N2) subclade J.2 and K (J.2.4.1) viruses, Hong Kong, 2025

**DOI:** 10.2807/1560-7917.ES.2026.31.10.2600185

**Published:** 2026-03-12

**Authors:** Samuel MS Cheng, Nancy HL Leung, Eunice Y Shiu, Ricky WK Wong, Niki YM Au, Xiaotong Huang, Natalie KM Kwok, Coco HC Chan, John KC Li, Leo CH Tsang, Tsz Chun Kwan, Tim K Tsang, Leo LM Poon, Benjamin J Cowling, Malik Peiris

**Affiliations:** 1 School of Public Health, The University of Hong Kong, Hong Kong Special Administrative Region, China; 2 Centre of Immunology and Infection, Hong Kong Science and Technology Park, Hong Kong Special Administrative Region, China; * These authors contributed equally to this work and share first authorship.; ** These authors contributed equally to this work and share last authorship.

**Keywords:** influenza A, subclade K, serology, cross-reaction, population immunity, vaccines, infection

## Abstract

We assessed haemagglutination inhibition (HAI) antibody titres against 2025 influenza A(H3N2) J.2 vaccine reference viruses and the novel subclade K (J.2.4.1) in age-stratified Hong Kong sera (collected May–July 2025), vaccine recipients and sera from virologically confirmed subclade J.2.2 or K infections. In contrast to mono-specific ferret sera, human sera with high J.2 HAI titres cross-reacted strongly with subclade K. Recombinant haemagglutinin- or egg-based standard dose J.2 A(H3N2) vaccines boosted geometric mean titres to K virus 5.6-fold and 2.6-fold, respectively.

Influenza A(H3N2) viruses within subclade K (formerly J.2.4.1) have caused extensive disease outbreaks globally, sometimes associated with atypical seasonal patterns [1-3]. Antigenic characterisation using mono-specific ferret antisera raised to northern hemisphere (NH) 2025/26 A(H3N2) vaccine reference strains (egg-propagated A/Croatia/10136RV/2023-like and cell-propagated A/District of Columbia/27/2023-like) indicated a marked reduction (≥ 32-fold) in cross-reactivity with subclade K viruses suggesting considerable antigenic drift [3].

We measured haemagglutination inhibition (HAI) antibody titres against the A(H3N2) NH 2025/26 egg- and cell-propagated J.2 vaccine reference viruses and a representative subclade K isolate, using: (i) age-stratified community sera collected in Hong Kong in 2025 before local influenza A(H3N2) subclade K circulation, (ii) pre-and-post vaccination sera in individuals immunised with recombinant protein-based (Flublok, Sanofi Pasteur) or egg-based standard-dose influenza A(H3N2) subclade J.2 vaccines, and (iii) paired sera from patients with RT-PCR-confirmed influenza A(H3N2) subclade J.2.2 or K infections.

## Study design

We used MDCK-SIAT1 cells or embryonated chicken eggs for virus isolation and propagation. Virus sequencing was carried out using the iSeq 100 System (Illumina). Sequence reads were demultiplexed and assembled using the United States Centers for Disease Control and Prevention (US CDC) MIRA pipeline. The HAI assay was performed in accordance with World Health Organization (WHO) guidelines [4]. Because adaptive mutations arise when influenza A(H3N2) viruses are grown in eggs, we used both egg-grown and cell-grown A(H3N2) virus antigens for serology assays. To assess whether neuraminidase binding to red blood cells contributes to the haemagglutinating activity of these virus antigens, we carried out parallel testing of a subset of sera with or without oseltamivir carboxylate. Inclusion of oseltamivir made no difference to the haemagglutinin (HA) titres of the antigens or HAI titres with the antisera (data not shown). Thus, we did not include oseltamivir in the HAI tests in the main study.

For this analysis, we used age-stratified sera collected from the healthy Hong Kong population as part an ongoing study [5] between May and July 2025, before the spread of subclade K in Hong Kong. Population immunity was estimated using a validated Bayesian framework [6]. Sera were stratified by age group and HAI titre level. We calculated two population immunity estimators: (i) the proportion of the population that is immune, weighted by age-stratified titre-specific protection probabilities, assuming 50% protection at titre 40; and (ii) the relative reduction in the basic reproduction number (R_0_), calculated using a next-generation matrix framework with age-specific susceptibility and social contact matrices [7]. The proportion of the population that is immune was weighted by the population age structure obtained from 2023 census data [8]. For the relative reduction in R_0_, we defined R_0_ as the largest eigenvalue of the contact matrix and calculated the effective reproduction number (Re) as the largest eigenvalue of the transmission matrix adjusted for susceptible population only. Estimates and 95% credible intervals (CrI) were obtained from 10,000 posterior samples. All analyses were performed using the ImmuPop R package [6].

## Pre-subclade K outbreak population HAI geometric mean titres 

**Figure 1:**
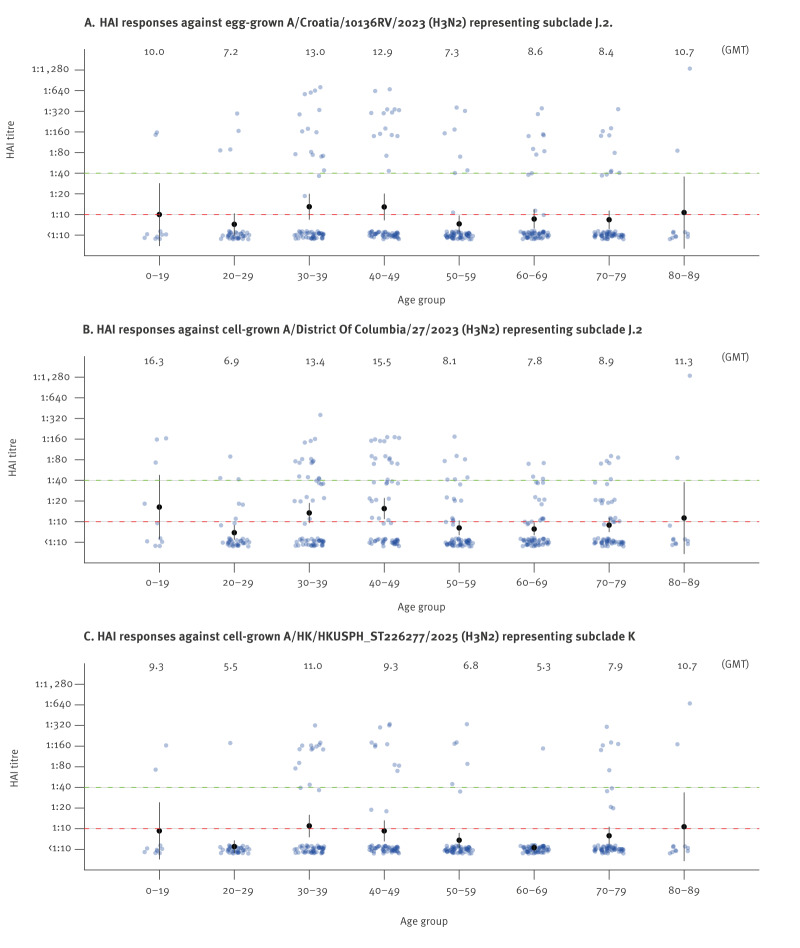
Age-stratified haemagglutination inhibition antibody titre distributions against influenza A(H3N2) virus subclades J.2 and K, Hong Kong, May–July 2025 (n =357)

GMT: geometric mean titre; HAI: haemagglutination inhibition.

Pre-subclade K outbreak population HAI geometric mean titres (GMTs) across different age strata against the egg-derived J.2 vaccine antigen, cell-derived J.2 antigen and cell-derived subclade K viruses ranged from 7.2 to 13.0, 6.9 to 16.3 and 5.3 to 11.0, respectively (Figure 1). Among 357 tested sera, 154 were from male and 203 were from female participants, with ages ranging from 5 to 85 years. Individuals with HAI titres of 40 to subclade K virus ranged from 1.7% in the 60–69-year age group to 25% in those aged 30–39 years.

Distribution of HAI antibody titres across eight age groups (0–19 (n=10), 20–29 (n=36), 30–39 (n=60), 40–49 (n=60), 50–59 (n=60), 60–69 (n=60), 70–79 (n=60) and 80–89 (n=11) years) for three influenza A(H3N2) antigens. Blue dots: individual serum samples; black dots and whiskers: GMT and 95% confidence intervals for each age group; green dashed line: protection threshold; red dashed line: detection threshold.

## Population immunity estimates against influenza A(H3N2) subclades 

**Figure 2:**
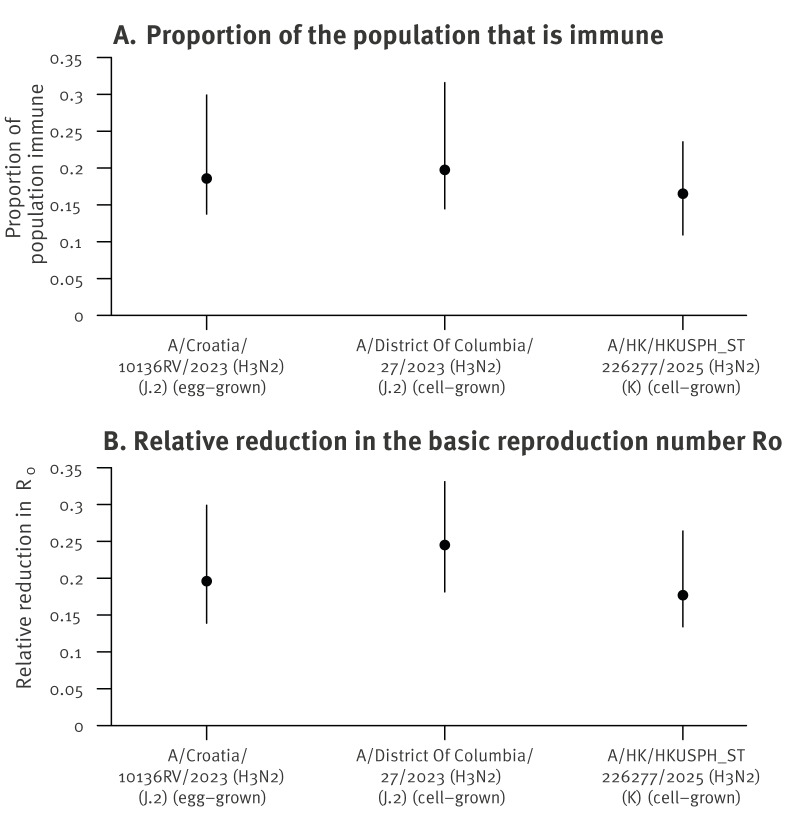
Population immunity estimates and predicted epidemiological impact against influenza A(H3N2) subclades, Hong Kong, May–July 2025 (n = 357)

The HAI titre is correlated with protection [9]. Our estimates of overall population immunity against different influenza A(H3N2) subclades and its potential effect on R_0_ are shown in Figure 2. After applying the protection associated with each HAI titre level and weighting by age using the population age structure, we estimated that 18.6% (95% CrI: 13.8–29.9) of the population was immune to egg-grown A/Croatia/10136RV/2023 (J.2), and 19.8% (95% CrI: 14.5–31.6) was immune to cell-grown A/District Of Columbia/27/2023 (J.2). In comparison, 16.5% (95% CrI: 10.9–23.6) of the population was immune to the cell-grown K subclade antigen A/HK/HKUSPH_ST226277/2025, representing a 16.7% lower point estimate of population immunity compared with the cell-grown J.2 variant. We estimated that the population immunity would reduce the R_0_ of egg-grown A/Croatia/10136RV/2023 (J.2) by 19.6% (95% CrI: 13.9–29.9), cell-grown A/District Of Columbia/27/2023 (J.2) by 24.5% (95% CrI: 18.1–33.1), and cell-grown A/HK/HKUSPH_ST226277/2025 (K) by 17.7% (95% CrI: 13.4–26.4). Comparing the two cell-grown antigens, there was a 27.8% lower point estimate of reduction in R_0_ for the K subclade compared with the J.2 subclade (Figure 2B). The reduction of population immunity to subclade K virus was lower that may be expected from the ≥ 4-fold reduction of HAI titres observed to mono-specific ferret immune sera [3].

Point estimates and 95% credible intervals are denoted. Higher values of these estimates indicate greater population-level protection.

To assess the extent of HAI cross-reactivity between A(H3N2) J.2 and K in human sera, we selected sera with HAI titres of 160–320 to cell-grown J.2 virus (n = 14) and assessed fold-reduction of HAI titres to subclade K virus. The GMT to subclade J.2 was 168.1, and the GMT to subclade K was 51.2, a 3.3-fold reduction. Nine of these 14 sera had only twofold or lower titres to the novel drift variant, one had an eightfold reduction and four had ≥ 32-fold reduction in HAI titres. There was no significant association between age and reduction in cross-reactivity using Mann-Whitney test, noting limited statistical power due to the small sample size.

## Homologous and heterologous haemagglutination inhibition boosting by influenza A(H3N2) clade J.2 vaccines

Pre- and post-vaccination sera in individuals immunised with recombinant protein-based (Flublok) (n=21; 9 females, 12 males; median age: 39.9 years, range: 24.4–49.8) or egg-based standard-dose influenza A(H3N2) subclade J.2 vaccines (Vaxigrip, Sanofi or Fluarix, GSK) (n=27; 15 females, 12 males; median age: 57.1 years, range: 26.6–81.9) were collected in Hong Kong between July and November 2025. 

**Figure 3:**
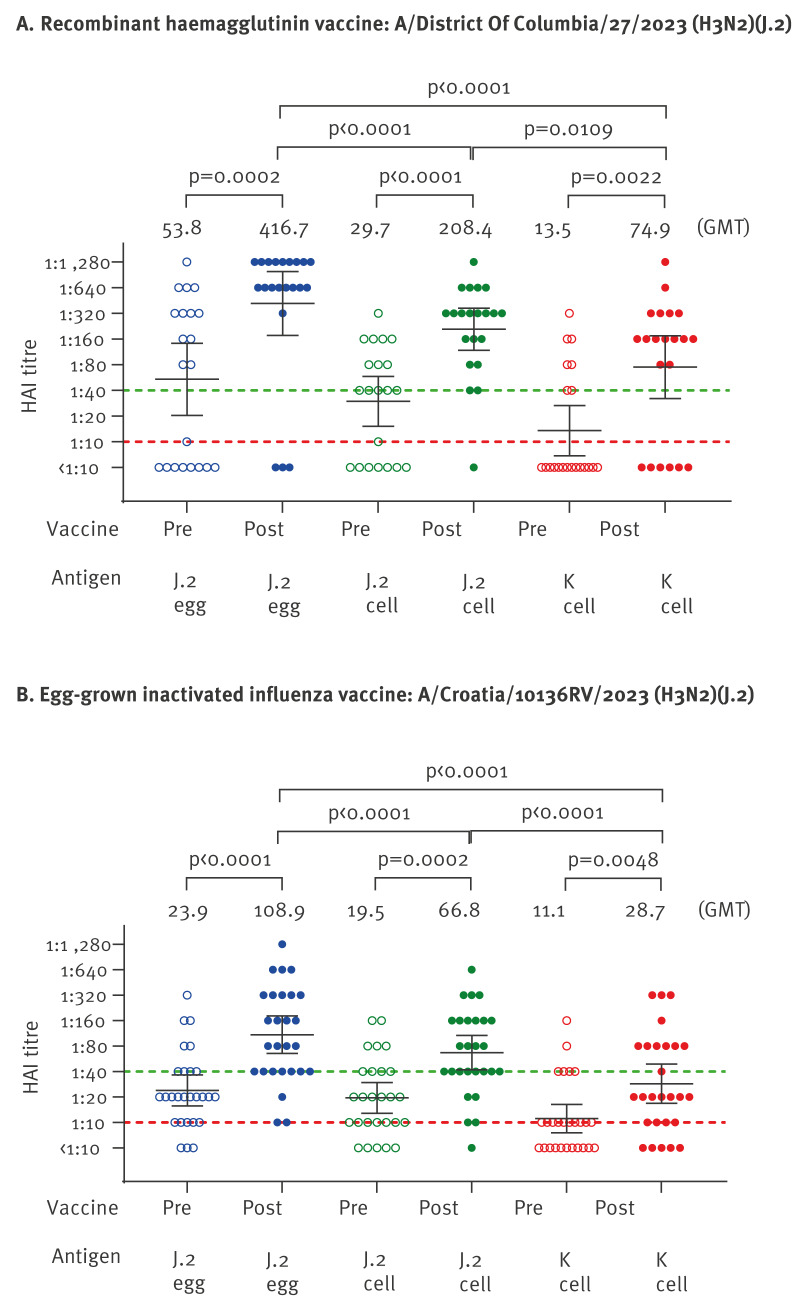
Haemagglutination inhibition titres measured before and 25–35 days after Flublok recombinant vaccine (n = 21) or egg-grown subunit influenza A(H3N2) subclade J.2 vaccines (n = 27), Hong Kong, July–November 2025

GMT: geometric mean titre; HAI: haemagglutination inhibition.

Flublok (influenza A(H3N2), subclade J.2) vaccine led to GMT increases of 7.8-fold (two-tailed Wilcoxon signed-rank test p = 0.0005), 7.0-fold (p < 0.0001) and 5.6-fold (p = 0.0001), to egg-grown subclade J.2 virus, cell-grown J.2 virus and cell-grown K virus, respectively. Post-vaccination HAI titres exceeded the threshold titre 40 in 18, 20 and 15 of the 21 participants vs egg-grown J.2 antigen, cell-grown J.2 antigen and cell-grown K antigen, respectively (Figure 3).

Each point represents an individual serum sample (open circles: pre-infection; filled circles: post-infection). Blue: responses to the egg-grown vaccine strain, green: to the cell-grown vaccine strain, red: to strain K. Black lines and whiskers: GMTs and 95% confidence intervals for each group; p values from two-tailed Mann–Whitney tests comparing pre- and post-infection titres are shown above the corresponding pairs. Wilcoxon test was used to compare HAI titre of the same sera against different viruses. Red dashed line: assay detection threshold (1:10), green dashed line: putative protective threshold (1:40).

Egg-grown inactivated influenza vaccines (Sanofi or GSK) led to GMT increases of 4.6-fold (p < 0.0001), 3.4-fold (p < 0.0001) and 2.6-fold (p = 0.001), to egg-grown J.2 virus, cell-grown J.2 virus and cell-grown K virus, respectively. Post-vaccination HAI titres exceeded the threshold titre 40 to these three antigens in 24, 22 and 11 of the 27 participants, respectively (Figure 3).

These data indicate strong homologous HAI boosting to J.2 antigens (both egg-grown and cell-grown) with lower but substantial cross-reactive responses to subclade K. Flublok vaccinees achieved higher post-vaccination GMTs than the egg-based Sanofi/GSK vaccinees against all three antigens: 416.7 vs 108.9 for J.2 (egg-grown), 208.4 vs 66.8 for J.2 (cell-grown) and 74.9 vs 28.7 for K (cell-grown). With both vaccines, use of cell-grown antigen for HAI assays resulted in lower GMTs than use of egg-grown HA antigen.

## Antibody responses following infection with influenza A(H3N2) subclade J.2.2 and K viruses

**Figure 4:**
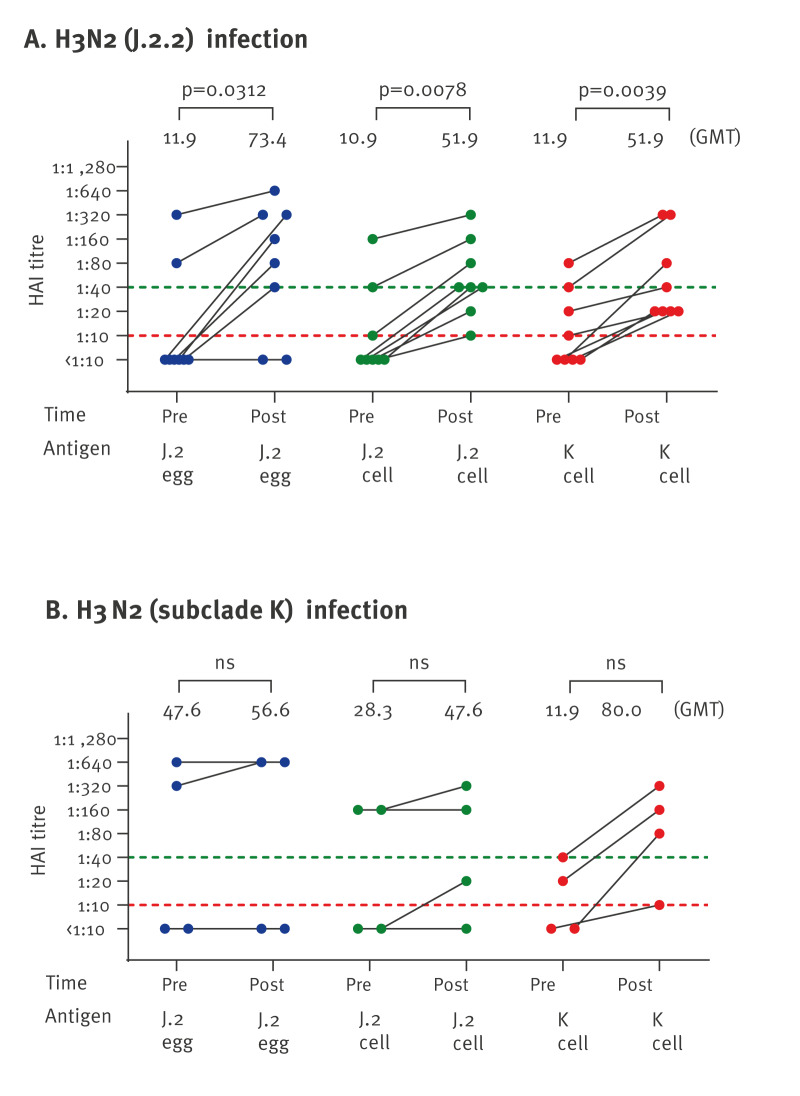
Haemagglutination inhibition titres measured before and 23–39 days after natural infection with influenza A(H3N2), Hong Kong, July–November 2025 (n = 12)

GMT: geometric mean titre; HAI: haemagglutination inhibition; ns: sot significant.

Paired sera from patients with RT-PCR-confirmed influenza A (H3N2) subclade J.2.2 (n = 8) or K infections (n = 4) were collected in Hong Kong between July and November 2025, with post-infection samples collected 23–39 days after symptom onset. Following A(H3N2) subclade J.2.2 infections (n = 8), HAI GMT titres increased 4.8-fold and 4.4-fold vs the homologous (J.2) and heterologous (K) cell-grown virus antigens, respectively; post-infection GMT was 51.9 to both cell-grown viruses (Figure 4). In subclade K infections (n = 4), HAI GMT increased 6.7-fold vs the homologous K virus and 1.7-fold vs the cell-grown J.2 virus antigens; post-infection GMT to homologous virus was 80.0, and 47.6 to J.2. Collectively, these data suggest that subclade J.2.2 virus infection elicited measurable cross-reactive responses to drifted subclade K, while there was less cross-reactive response to J.2 virus from subclade K infection.

Each point represents an individual serum sample. Paired pre- and post- infection sera of the same individual are connected by a line. Blue: responses to the egg-grown vaccine strain, green: to the cell-grown vaccine strain, red: to strain K. GMTs are indicated above each group; p values from two-tailed Wilcoxon tests comparing pre- and post-infection titres are shown above the corresponding pairs. Red dashed line: assay detection threshold (1:10), green dashed line: putative protective threshold (1:40).

## Discussion

Our data suggest that the Hong Kong population in mid-2025 had low levels of HAI titres to both subclade J.2 and K virus antigens. It was therefore not surprising that Hong Kong was affected by a large influenza A(H3N2) subclade K virus outbreak in late 2025, although the magnitude of this outbreak was not markedly larger than the preceding influenza A(H1N1)pdm09 epidemic waves in 2024 and early 2025 [10].

Although mono-specific ferret sera had indicated very low cross-reactivity (≥ 32-fold titre difference) between subclade J.2 and K viruses, we observed greater cross-reactivity with sera from human patients, who have a more complex influenza A(H3N2) infection history. The GMT reduction in sera from the Hong Kong population was only 3.3-fold, the GMT reduction in recombinant vaccine recipients was 2.8-fold and in conventional egg-grown sub-unit vaccines only 2.3-fold. Sera from humans recently infected with J.2.2 A(H3N2) viruses had comparable GMT to subclade J.2 or K. These data accord with observations that real-world vaccine effectiveness of J.2-based vaccines against influenza-related disease and hospital admission remained within typical ranges for influenza A(H3N2) seasons in Europe during a period when subclade K predominated [2,11]. The WHO has advised that vaccination remains important for preventing severe outcomes [1]. Our data highlight the importance of using sera from human populations to complement results from mono-specific ferret sera when assessing the potential impact of a novel variant. A previous study that comprehensively compared breadth of cross-reactivity of mono-specific ferret sera and human sera also concluded that adult human sera had broader cross-reactivity to future drift variants compared with mono-specific ferret sera [12].

Our results show significant differences in antibody titres when cell-grown or egg-grown viruses are used as antigens for HAI tests. The data also highlight better immunogenicity elicited by the recombinant vaccine which has HA antigens not affected by passage in embryonated eggs. The higher titres to the homologous cell-grown J.2 antigens also reflect higher cross-reactive titres to the drift variant K.

The WHO updated the influenza A(H3N2) components for the 2026 southern hemisphere vaccine to J.2.4-lineage viruses [13], further illustrating a need for continued antigenic monitoring as subclade K and related viruses may diversify. 

Our study has limitations. The number of paired sera from RT‑PCR–confirmed influenza A(H3N2) subclade J.2.2 or K infections was small (< 20 individuals). These constraints limited statistical power to characterise infection‑induced antibody dynamics by subclade and increase uncertainty around estimates of cross‑reactive boosting. The findings should therefore be interpreted with caution pending larger, longitudinal datasets.

## Conclusion

Human serology revealed broader cross‑reactivity to the emergent influenza A(H3N2) subclade K than suggested by mono‑specific ferret antisera, with community sera post‑vaccination or post-infection showing measurable inhibition of K despite antigenic drift from J.2. Both recombinant protein-based and standard‑dose egg-based J.2 vaccines elicited strong homologous responses and boosted cross‑reactive antibodies to K, with recombinant protein-based vaccine achieving the highest cross‑reactive titres. Nevertheless, mid-2025 population immunity to both J.2 and K was low, consistent with susceptibility to the outbreak in late 2025. These findings support integrating human serology alongside ferret data in antigenic risk assessment and vaccine‑strain deliberations. While vaccine updates are pursued, current J.2‑based vaccines can still enhance cross‑reactive immunity and may mitigate immediate transmission risk. 

## Data Availability

The complete genome sequences for A/HK/HKUSPH_ST226277/2025 (subclade K) are available in GenBank (accession number PX700687). Other data are available upon request.
